# Pediatric Adrenal Insufficiency: Diagnosis, Management, and New Therapies

**DOI:** 10.1155/2018/1739831

**Published:** 2018-11-01

**Authors:** Sasigarn A. Bowden, Rohan Henry

**Affiliations:** Division of Endocrinology, Department of Pediatrics, Nationwide Children's Hospital/The Ohio State University College of Medicine, Columbus, Ohio, USA

## Abstract

Adrenal insufficiency may result from a wide variety of congenital or acquired disorders of hypothalamus, pituitary, or adrenal cortex. Destruction or dysfunction of the adrenal cortex is the cause of primary adrenal insufficiency, while secondary adrenal insufficiency is a result of pituitary or hypothalamic disease. Timely diagnosis and clinical management of adrenal insufficiency are critical to prevent morbidity and mortality. This review summarizes the etiologies, presentation, and diagnosis of adrenal insufficiency utilizing different dynamic hormone testing and describes current treatment recommendations and new therapies.

## 1. Introduction

Adrenal Insufficiency (AI) may be caused by destruction or dysfunction of the adrenal gland (primary AI, Addison's disease), deficient pituitary adrenocorticotrophic hormone (ACTH) secretion (secondary AI), or deficient hypothalamic secretion of corticotropic releasing hormone (CRH) (tertiary AI). The secondary and tertiary AI can also be called central AI. Primary AI is rare with a prevalence of approximately 93-140 per 1,000,000 [1–3]. The most common cause of primary AI in children is congenital adrenal hyperplasia (CAH) which accounts for 70% of pediatric patients with primary AI, whereas autoimmune adrenalitis (Addison's disease) accounts for up to 15% of cases [4]. The most common cause of CAH is 21-hydroxylase deficiency, accounting for ~ 90% of all CAH cases, with an incidence of 1 in 14,000 live births [5]. Secondary AI secondary to intracranial pathology is also rare and may be isolated deficiency of ACTH or CRH, or it may be part of other pituitary hormonal deficiencies, called hypopituitarism. Iatrogenic tertiary AI caused by suppression of the hypothalamic-pituitary adrenal (HPA) axis secondary to glucocorticoid administration is the most common cause of central AI, with an estimated prevalence of 150- 280 per 1,000,000 [6]. AI is associated with considerable morbidity and mortality [7], frequently associated with lack of awareness or education regarding AI management, especially at time of physical stress that requires increased GC dose. This review summarizes the etiologies, presentation, diagnosis, and treatment of AI and highlights new therapies.

## 2. Etiology and Presentation of Adrenal Insufficiency

### 2.1. Primary AI

The most common cause of primary AI in children is CAH, which is due to a deficiency of one of several enzymes required for adrenal synthesis of cortisol. CAH may be associated with aldosterone deficiency or excess, depending on the type of enzyme deficiency. More commonly, CAH can also be associated with androgen excess but can also be in combination with androgen deficiency. For all other causes of primary AI, all 3 zones of the adrenal cortex are usually involved by a disease process. The process can be local or a manifestation of systemic disorders, such as autoimmune disease (either isolated or as part of the polyglandular autoimmune syndrome), granulomatous diseases such as tuberculosis and histoplasmosis, and hemorrhage associated with meningococcemia. Primary AI can also be due to rare genetic diseases, which include disorders of steroidogenesis, peroxisomal defects, and abnormal adrenal gland development due to mutations (Table 1). The result is inadequate secretion of glucocorticoids, mineralocorticoids, and androgens.

**Figure 1:**
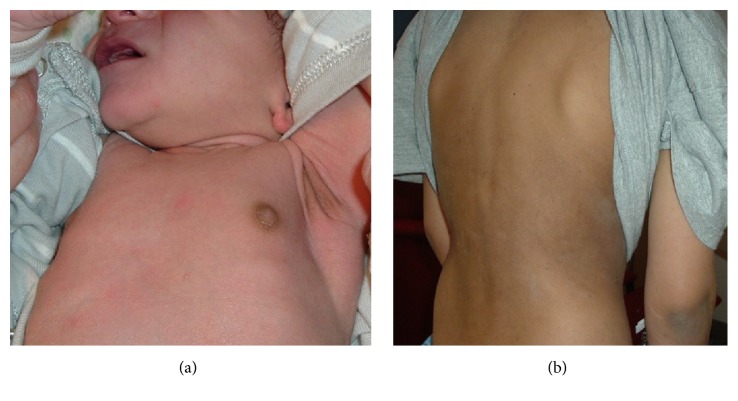
(a) Hyperpigmentation at nipples and axillar in a 10-day-old male infant with salt-wasting congenital adrenal hyperplasia due to 21-hydroxylase deficiency. (b) Hyperpigmentation in a 9-year-old boy with Addison's disease.

The clinical presentation of AI can be gradual and nonspecific to hypotension or shock, so-called adrenal crisis, depending on the degree of insufficiency and precipitating stress events. Symptoms include fatigue, nausea, muscle weakness, and headache. Infants with salt wasting CAH often present in the second week of life with signs of acute primary AI which include dehydration, weight loss, lethargy, hyponatremia, hyperkalemia, and hypoglycemia. In older children and adolescents, symptoms may include fatigue, nausea, vomiting, diarrhea, abdominal pain, weight loss, slow growth, and salt craving. The lack of cortisol negative feedback increases hypothalamic CRH, leading to increased pituitary ACTH and melanocyte-stimulating hormone (MSH), both of which are derived from a precursor, proopiomelanocortin (POMC). When CRH is cleaved from POMC, MSH is concurrently released. Elevated ACTH and MSH cause hyperpigmentation of the skin (Figure 1) and mucous membranes, involving skin creases, axillae, groin, gingival, and scars.

Aldosterone deficiency causes sodium loss, which leads to electrolyte abnormalities including hyponatremia, hyperkalemia, and metabolic acidosis. Symptoms of aldosterone deficiency include salt craving, anorexia, dizziness, hypotension, dehydration, and weight loss.

### 2.2. Secondary AI

Secondary AI or central AI is caused by deficiency of pituitary ACTH or hypothalamic CRH secretion and consequent insufficient adrenal cortisol secretion (Table 1). Clinical presentation of central AI is similar to that of primary AI but without salt wasting, because aldosterone secretion is normal in central AI, being regulated by the renin-aldosterone pathway. Therefore, dehydration, hypotension, hyponatremia, and hyperkalemia are usually not present. With the absence of an increased production of ACTH, patients with secondary AI do not have hyperpigmentation. In isolated ACTH deficiency or in combination with growth hormone deficiency as part of hypopituitarism, hypoglycemia can occur which can lead to seizures and coma if severe [8].

## 3. Diagnosis of AI

Because the signs and symptoms of AI are nonspecific, clinicians must have high index of suspicion. Serum electrolytes often provide a clue to diagnosis, as hyponatremia with or without hyperkalemia is common in patients with primary AI. Hyponatremia with absence of hyperkalemia in primary AI can be explained by protracted vomiting, a common symptom at presentation [9]. Hyponatremia is very common in primary AI due to aldosterone deficiency. However, hyponatremia can also be seen in patients with central AI. The explanation for this is vasopressin hypersecretion with resultant water retention [10]. The lack of cortisol negative feedback not only increases hypothalamic CRH but also increases vasopressin synthesis and secretion. Hypoglycemia can be seen in both primary and secondary AI and can be more pronounced in secondary AI in combination with growth hormone deficiency. Hypoglycemia may be a presenting symptom; therefore, laboratory investigation for hypoglycemia should include serum cortisol, drawn at the time of hypoglycemia. Patients with AI have impaired gluconeogenesis and hepatic glycogenesis; therefore, hypoglycemia may be associated with ketosis.

In primary AI with salt losing crisis, plasma renin activity is elevated, while aldosterone secretion is low. Urinary excretion of sodium and chloride is increased and that of potassium decreased.

The most definitive test is measurement of serum cortisol levels. A morning cortisol of < 3 ug/dL is indicative of adrenal insufficiency, while cortisol level >18 mcg/dL rules out adrenal insufficiency. A diagnosis of primary AI is confirmed if the serum cortisol level is < 18 mcg/dL, in the presence of markedly elevated ACTH and plasma renin activity. It is important to note that the cortisol level cutoff may differ among different laboratories, depending on the assays by which cortisol is measured [11]. Conditions affecting cortisol-binding globulin (estrogen hormone, as in pregnancy or the use of oral contraceptives or hypoproteinemia such as nephrotic syndrome) may also affect cortisol values [12].

Positive adrenal autoantibodies establish autoimmune adrenal insufficiency or Addison's disease. All males diagnosed with primary adrenal insufficiency without evidence of autoimmunity should have plasma very long chain fatty acids obtained to rule out X-linked adrenoleukodystrophy.

When CAH is being considered in newborns presenting with ambiguous genitalia or salt-losing crisis, random cortisol and androgen hormone studies, particularly 17-hydroxy progesterone, are obtained to confirm or exclude diagnosis. Patients with mild or early stage of AI or central AI often require additional dynamic testing.

## 4. Dynamic Testing to Assess the HPA Axis

### 4.1. ACTH Stimulation Test

Administration of cosyntropin (1-24 ACTH; Cortrosyn) to directly stimulate adrenal cortisol release is the most commonly used diagnostic test to evaluate adrenal function. Baseline ACTH and cortisol samples are obtained (with additional tests such as plasma renin activity, aldosterone, or androgen hormones as indicated), and then 250 *μ*g of cosyntropin is administered intravenously, followed by cortisol samples drawn at 30 and 60 minutes later. Plasma cortisol level ≥ 18 *μ*g/dL, along with a normal baseline ACTH level rules out primary adrenal insufficiency. This test may not be sensitive in identifying patients with mild AI or recent onset secondary adrenal insufficiency [13] as adrenal reserve may still be adequate with a normal cortisol response to exogenous ACTH. Therefore, a low dose ACTH stimulation test using 1 *μ*g cosyntropin should be used in patients suspected of secondary AI as 1 *μ*g dose is more sensitive to detect AI, thereby preventing false positive results [14, 15]. Technical difficulties in performing the low dose ACTH stimulation test exist. These include dilution error of ACTH, afternoon testing, or loss of ACTH due to adherence to long plastic tubing through which ACTH is administered [16]. Hence, the clinician should be cognizant of these issues when interpreting test results.

In patients with central AI, ACTH level can be low or low normal. When the diagnosis of ACTH deficiency is made, it is important to rule out other pituitary hormone deficiencies because isolated ACTH deficiency is rare.

In neonates with congenital hypopituitarism, adrenal function test using 1 *µ*g ACTH stimulation test, performed during postnatal period to diagnose ACTH deficiency, may be falsely normal [17]. Newborns with ACTH deficiency have a normal upregulation of fetal steroidogenic enzymes and normal fetal adrenal maturation and steroidogenesis under the placental CRH stimulation [18], therefore, still have adequate adrenal reserve that allows temporary normal cortisol response to synthetic ACTH injection after birth. Clinicians must maintain a high degree of suspicion for false negative testing and repeat ACTH stimulation test within 3-4 weeks after initial testing, for timely diagnosis of central AI in these infants [17].

### 4.2. Glucagon Stimulation Test

Glucagon stimulation test is a sensitive test for evaluating adrenal function and is not associated with hypoglycemia and, therefore, provides an alternative to insulin-induced hypoglycemia in evaluating central hypoadrenalism. In this test, glucagon at 0.03 mg/kg (maximum 1 mg) is given subcutaneously. Blood samples for serum glucose and cortisol are obtained at 60, 90, 120, and 150 minutes after glucagon administration. Glucagon administration causes an increase in blood glucose which then evokes an endogenous insulin response, resulting in a fall in blood glucose which stimulates a counter-regulatory hormone response including cortisol [19]. Glucagon stimulation test was found to have a high false-positive rate of 23.7% in children (those who failed the test had a normal peak cortisol on the ACTH stimulation test) [20]. Moreover, peak glucagon-stimulated cortisol level was inversely related to age [20, 21] and gender [20] in children. Therefore, interpretation of glucagon stimulation test can be problematic. Lower peak cortisol cutoff for glucagon stimulation test has been suggested in adults [22, 23]. Glucagon-stimulated cortisol cutoff has not been established in pediatrics.

### 4.3. Insulin-Induced Hypoglycemia

Hypoglycemia provokes counter-regulatory hormone response and is used to assess the integrity of the HPA axis. This test was once considered the gold standard for the diagnosis of AI but is no longer used in children because of the risk of hypoglycemic seizures and severe hypokalemia after treatment with glucose infusion [7, 24].

### 4.4. Metyrapone Test

Metyrapone inhibits the activity of 11*β*-hydroxylase enzyme that converts precursor to cortisol, resulting in decreased cortisol secretion and a compensatory increase in ACTH levels, as well as 11-deoxy-cortisol (the precursor of cortisol) and its urinary metabolites. For the convenient single dose test, 30 mg/kg to a maximum of 3 grams is given at midnight with a snack to decrease the nausea associated with metyrapone ingestion. Cortisol, 11-deoxycortisol, and ACTH are measured at 8 AM following the dose. A normal response is the increase in plasma 11-deoxycortisol to > 7 *μ*g/dL [25]. Lack of increase in ACTH and 11-deoxycortisol levels after metyrapone administration is diagnostic of ACTH deficiency. The metyrapone test is an excellent test to evaluate the integrity of adrenal function but is rarely performed because of the difficulty in obtaining metyrapone and the risk of precipitating an adrenal crisis [7].

## 5. Treatment of AI

### 5.1. Maintenance Therapy

In primary adrenal insufficiency, maintenance therapy requires both glucocorticoids and mineralocorticoid replacement. In secondary or central adrenal insufficiency, only cortisol replacement is required without the need of salt-retaining aldosterone replacement.

#### 5.1.1. Hydrocortisone

The daily basal cortisol production rate in children is approximately 6-8 mg/m2/day, lower than previously estimated [26]. When administering orally, the recommended physiologic replacement dose of hydrocortisone in pediatric patients is approximately 10-12.5 mg/m2/day divided into two or three doses, compensating for the incomplete intestinal absorption and hepatic metabolism [7]. In children with AI secondary to CAH, a supraphysiologic dose of 12-20 mg/m2/day is required to suppress adrenal androgens. Goal of therapy is to control the symptoms of AI with the lowest dose possible, without compromising growth that is seen in overtreatment. Hydrocortisone is preferred in children over other types of glucocorticoid because it is easy to titrate and has a short half-life with less adverse effects, compared with the more potent longer-acting glucocorticoids.

#### 5.1.2. Hydrocortisone Dosing Consideration

When hydrocortisone is given twice a day in children and adolescents with hypopituitarism, a nonphysiological nadir of cortisol levels is observed 2-4 hours prior to the next dose [27]. Therefore, children with central AI (secondary to hypopituitarism) who are more prone to hypoglycemia or children with CAH who have additional risk of hyperandrogenism when hydrocortisone is inadequate should receive hydrocortisone 3 times a day. It is recommended that glucocorticoid should be administered with food to prolong the half-life of hydrocortisone and to facilitate the production of a more physiological cortisol profile [28].

In patients with both TSH and ACTH deficiency or patients with autoimmune polyendocrine syndrome type II (primary hypothyroidism and Addison's disease), treatment with levothyroxine may precipitate an acute adrenal crisis because thyroxine increases cortisol metabolism [29, 30]. Therefore, cortisol replacement should precede thyroid hormone replacement.

Although commercial liquid preparations of hydrocortisone are not recommended due to the uneven distribution of the drug in the liquid, hydrocortisone oral compounded suspension prepared extemporaneously from hydrocortisone tablets can be used safely and effectively in young children [31, 32]

Hydrocortisone dose needs to be increased to ensure adequate cortisol replacement when taken with medications that induce hepatic cortisol metabolism by enzyme induction of cytochrome P450 3A4. These medications that accelerate hepatic glucocorticoid metabolism include rifampicin, mitotane, anticonvulsants such as phenytoin, carbamazepine, oxcarbazepine, phenobarbital, and topiramate. Conversely, patient treated with drugs that inhibit CYP3A4 such as antiretroviral medication may require reduction of hydrocortisone dose [33].

#### 5.1.3. Other Glucocorticoids (Dexamethasone and Prednisone)

Dexamethasone can be used to treat patients with suspected AI while undergoing a diagnostic ACTH stimulation test, because the cortisol tests can be performed without the interference of dexamethasone. However, the use of dexamethasone for this purpose may suppress the HPA axis that may influence adrenal function testing. For long-term maintenance therapy in growing children, dexamethasone and prednisone are not recommended due to concerns of growth suppression and significant weight gain. In some circumstances when adherence to hydrocortisone is problematic, one can use oral prednisolone or prednisone that can be dosed every 12 hours. The conversion of hydrocortisone to prednisone is a 5:1 ratio (prednisone or prednisolone is 5 times more potent than hydrocortisone).

#### 5.1.4. Fludrocortisone

In children with primary AI and confirmed aldosterone deficiency, treatment with fludrocortisone at 0.05-0.2 mg per day in two divided doses is recommended. It has been suggested that fludrocortisone has not only mineralocorticoid but also potent glucocorticoid activity [9]. This is of particular relevance in newborns and infants to avoid glucocorticoid overexposure. The dose adjustment of fludrocortisone for body size is rarely required since the aldosterone secretion rate does not increase from infancy to adulthood. Excessive fludrocortisone can cause hypervolemia, hypertension, and edema. Monitoring of growth, weight gain, symptoms of salt craving, blood pressure, serum electrolytes, and plasma renin activity provides guidance for adjusting doses of fludrocortisone.

#### 5.1.5. Salt Supplementation

Because of low salt content in breast milk and infant formulas and mineralocorticoid resistance in the immature infant kidney, sodium chloride supplements at 1–2 gm/day (17–34 mEq per day) distributed in several feedings are given in the newborn period and up to the age of 8-12 months when salt intake from diet is sufficient [5, 34].

### 5.2. Acute Management of AI or Adrenal Crisis

Adrenal crisis is a life-threatening emergency that requires prompt diagnosis and treatment. Acute AI must be treated urgently with sufficient parenteral hydrocortisone, 100-150 mg or 100 mg per m2 intravenously, saline with dextrose to restore intravascular volume, and normalized serum sodium and blood glucose concentrations. Treatment of underlying conditions such as infection or trauma must also be undertaken. An intravenous isotonic saline with dextrose infusion at maintenance rate should be continued for the following 24-48 hours until patient is hemodynamically stable. Intravenous hydrocortisone at stress dose (100 mg per m2/day) given as continuous infusion or intravenous boluses every 6 hours should be continued in the first 24 hours and tapered over 2-3 days (if clinically stable) to oral glucocorticoid maintenance dose. Mineralocorticoid replacement with fludrocortisone should be started in patients with primary AI when they are able to have oral intake.

### 5.3. Treatment during Illness, Injury, or Surgery

Cortisol is an important stress hormone that is essential for human survival, particularly during stress. Surgery, anesthesia, trauma, and illnesses result in increased plasma ACTH and cortisol levels. Many studies have demonstrated increased daily cortisol secretion proportionate to the degree of stress in healthy adults undergoing surgery or in acutely ill individuals [35, 36]. According to the recommendations published by the Pediatric Endocrine Society Drug and Therapeutic Committee, the stress hydrocortisone doses are 30-50 mg/m2/day for mild to moderate stress and 100 mg/m2/day for the most severe stresses, such as major surgery or critical illness [7]. The initial dose is followed by the same dose at a constant rate over a 24-hour period. The stress doses of hydrocortisone are tapered back to physiologic dose based on the pace of clinical improvement usually within 2-3 days. Patients with diarrhea and vomiting, who are unable to take oral medication by mouth, require intramuscular hydrocortisone (100 mg/m2 per dose). In an emergency event when patient's weight or height may not be available, a quick, simple age-based dosing can be used: 25 mg IV/IM for 0-3 years, 50 mg for 3-12 years, and 100 mg for ≥12 years.

### 5.4. Novel Therapies

The current oral glucocorticoid replacement therapy for patients with AI does not truly mimic the normal physiologic cortisol rhythm, a nadir at bedtime and gradually rising levels to the early morning peak between 3 am and 6 am before waking. Many patients continue to have fatigue, nausea, and headaches on current conventional therapy [37] and some have nocturnal hypoglycemia due to very low cortisol levels during the night and early morning [38]. Moreover, a higher prevalence of obesity, impaired glucose tolerance, and dyslipidemia has been demonstrated in patients with Addison's disease [39], with increased use of antihypertensive drugs and lipid-lowering agents, as well as an increased risk for cardiovascular morbidity compared to general population [40]. Children with CAH, on supraphysiological glucocorticoid doses that cause an altered diurnal cortisol profile, have high rate of obesity, hypertension, growth suppression, and low bone density [41–43]. Increased evening cortisol levels have been shown to reduce glucose tolerance, insulin secretion, and insulin sensitivity in healthy young adults [44]. Because of all these concerns and unfavorable treatment outcomes, novel therapies have been developed in recent years.

#### 5.4.1. Continuous Subcutaneous Hydrocortisone Infusion

Continuous subcutaneous hydrocortisone infusion therapy administered via an insulin pump has been utilized to deliver hydrocortisone. Several studies have demonstrated that this mode of drug delivery restores a circadian cortisol rhythm and normalizes the ACTH levels compared to conventional therapy [45] and improves quality of life [46]. However, due to high cost and other risks associated with the use of pump (such as site and pump failures), continuous subcutaneous hydrocortisone infusion has not been used routinely in clinical practice but can be considered a treatment option in classic CAH poorly controlled on conventional therapy [47].

#### 5.4.2. Sustained Release Hydrocortisone Preparations

Three modified release hydrocortisone formulations have been developed in Europe to mimic a normal circadian rhythm of cortisol. Chronocort is a hydrocortisone preparation with delayed-release given twice a day, with a larger dose given at night before sleep and a smaller dose given in the morning. The large dose given at night is to suppress the overnight ACTH surge which drives excess androgen production in CAH and to provide a high peak cortisol early in the morning at wakening. Studies in adults with CAH resulted in more physiologic cortisol profile similar to that seen in healthy individuals. Moreover, patients on modified release hydrocortisone preparation had a better control of androgen levels throughout the day [48, 49].

A dual-release hydrocortisone preparation is given once daily in the morning, with immediate release coating that is rapidly absorbed, followed by a slow release from the core of tablet. Studies of this hydrocortisone formulation in adults with Addison's disease have shown to achieve physiologic cortisol profile, reduce central adiposity, and improve metabolic parameters, as well as quality of life [50, 51]. Once-daily, modified–release hydrocortisone treatment in patients with AI restores a more physiologic circadian cortisol rhythm, normalizes the immune cell profile, and reduces recurrent infections, compared to treatment with conventional glucocorticoid replacement therapy [52].

Infacort is an immediate-release, oral formulation of hydrocortisone which has been designed specifically for infants and children. Infacort is provided in capsules containing taste-masked granules or sprinkle, which allows flexible low dosing to children in units of 0.5 mg, 1 mg, 2 mg, and 5 mg of hydrocortisone [53]. Infacort has been shown to be easy to administer to neonates, infants, and children with good absorption, achieving cortisol levels at 60 minutes after administration similar to physiologic cortisol levels in healthy children [53].

## 6. Patient Education and Emergency Precautions

Education of the patient and family is the key to successful therapy of AI and prevention of morbidity and mortality associated with AI. The patient and their caretakers must be instructed about the rationale for replacement therapy, the maintenance medications, and stress dosing for illnesses. They need to be taught on how to administer injectable glucocorticoid when the patient is vomiting or unable to take oral stress doses, and when to consult a physician or go to the emergency department. All patients should at all times wear a medical alert identification and carry a medical emergency information card that indicate the diagnosis of “adrenal insufficiency” and daily medications.

## Figures and Tables

**Table 1:** Causes of adrenal insufficiency.

PRIMARY
(i) Congenital adrenal hyperplasia
(ii) Congenital adrenal hypoplasia due to gene mutations (e.g. DAX-1, SF1 mutations)
(iii) Peroxisome defects (adrenoleukodystrophy [childhood or neonatal], Zellweger syndrome)
(iv) Bilateral adrenal hemorrhage of the newborn
(v) Adrenal hemorrhage of acute infection (Waterhouse-Friderichsen syndrome)
(vi) Autoimmune adrenalitis (isolated or part of autoimmune polyglandular syndrome type 1 and 2)
(vii) Infection (e.g. tuberculosis, fungal infection, human immunodeficiency virus, cytomegalovirus)
(viii) Triple A syndrome or Allgrove syndrome (alacrimia, achalasia, adrenal insufficiency)
(ix) Adrenal unresponsiveness to ACTH due to gene mutations
(x) Familial glucocorticoid deficiency
(xi) Drug effects (mitotane, ketoconazole, aminoglutethimide, metyrapone, megestrol, rifampin)
SECONDARY (CENTRAL)
(i) Congenital
(a) Septo-optic dysplasia
(b) Pituitary aplasia/hypoplasia
(c) Agenesis of corticotrophs
(d) POMC
(ii) Acquired
(a) Trauma
(b) Brain tumor (craniopharyngioma)
(c) Lymphocytic hypophysitis
(d) Surgery
(e) Cranial irradiation
(f) Infiltrative disease (hemochromatosis, sarcoidosis, Langerhans cell histiocytosis)
(g) Steroid withdrawal after prolonged administration
